# Medically Useful Plant Terpenoids: Biosynthesis, Occurrence, and Mechanism of Action

**DOI:** 10.3390/molecules24213961

**Published:** 2019-11-01

**Authors:** Matthew E. Bergman, Benjamin Davis, Michael A. Phillips

**Affiliations:** 1 Department of Cellular and Systems Biology, University of Toronto, Toronto, ON M5S 3G5, Canada; matthew.bergman@mail.utoronto.ca (M.E.B.); benjamin.davis@mail.utoronto.ca (B.D.); 2 Department of Biology, University of Toronto–Mississauga, Mississauga, ON L5L 1C6, Canada

**Keywords:** isoprenoids, plant natural products, terpenoid biosynthesis, medicinal plants, terpene synthases, cytochrome P450s

## Abstract

Specialized plant terpenoids have found fortuitous uses in medicine due to their evolutionary and biochemical selection for biological activity in animals. However, these highly functionalized natural products are produced through complex biosynthetic pathways for which we have a complete understanding in only a few cases. Here we review some of the most effective and promising plant terpenoids that are currently used in medicine and medical research and provide updates on their biosynthesis, natural occurrence, and mechanism of action in the body. This includes pharmacologically useful plastidic terpenoids such as *p*-menthane monoterpenoids, cannabinoids, paclitaxel (taxol^®^), and ingenol mebutate which are derived from the 2-*C*-methyl-d-erythritol-4-phosphate (MEP) pathway, as well as cytosolic terpenoids such as thapsigargin and artemisinin produced through the mevalonate (MVA) pathway. We further provide a review of the MEP and MVA precursor pathways which supply the carbon skeletons for the downstream transformations yielding these medically significant natural products.

## 1. Plant Terpenoids

Terpenoids, or isoprenoids, are isoprene-based natural products with fundamental roles in the metabolism of all organisms [1]. Terpenoid chemical diversity is especially high in plants where many can be considered secondary metabolites. Such non-essential, specialized plant terpenoids underlie many ecological interactions between plants and animals [2,3], acting as allelochemicals to attract pollinators, repel herbivores, or attract herbivore predators [4]. The evolution of terpenoid secondary metabolism in plants began with the recruitment of genes from primary metabolism [5] and accelerated due to the proliferation of cytochrome P450 and terpene synthase gene families in the genomes of plants [6,7]. Terpenoid chemical diversity partly reflects a natural history marked by herbivory stress and other selective pressures imposed by animals, resulting in a broad array of functionalized terpenoids in the plant kingdom pre-selected for their potent biological activities towards animals [8]. This selective process may have been facilitated by the general similarity of protein folds and motifs between plant and animal proteins, resulting in plant secondary metabolites with natural affinity for animal proteins by virtue of having been produced by plant enzymes composed of the same amino acids [9]. Thus, pre-selection and emergence of plant secondary metabolites with biological activity towards animals may have both an evolutionary and a biochemical basis.

As a consequence, many plant terpenoids have found fortuitous uses in medicine, and the terpenoid family of natural products has been a valuable source of medical discoveries [10,11,12]. Hundreds more allegedly possess medicinal properties, but the testing process is painstaking and resource intensive. The true number of plant terpenoids in nature which could potentially be screened for therapeutic applications is unknown but is likely >10^5^, including >12,000 from the diterpenoid group alone [13]. While this number is small compared to modern combinatorial methods, the lead compound discovery rate may be significantly higher for plant natural products due to the aforementioned pre-selection effects. But owing to their high degree of chemical functionalization and metabolic specialization, many are produced in small amounts, only in response to elicitation, or accumulate exclusively in specialized tissues, necessitating microbial production or major advances by plant breeding and genetic improvement to obtain sufficient quantities to investigate clinical potential [14,15]. This in turn necessitates a detailed understanding of the biosynthetic enzymes, genes, and regulatory programs of a given candidate compound. Here we review the current understanding of the biosynthesis, occurrence, and mechanism of action of a select group of plant terpenoids of medical significance. Due to the large number of plant terpenoids described in the literature in various health contexts, we have excluded those of a purely nutritional, nutraceutical, or anti-oxidant nature and instead focused on compounds with specific pharmacological activity. Cardenolides such as digoxin, which inhibit cardiac Na^+^/K^+^ ATPase and improve cardiac muscle contraction and stroke volume, have been reviewed recently [16,17] and therefore were not covered here.

## 2. Terpenoid Precursor Pathways

**Figure 1:**
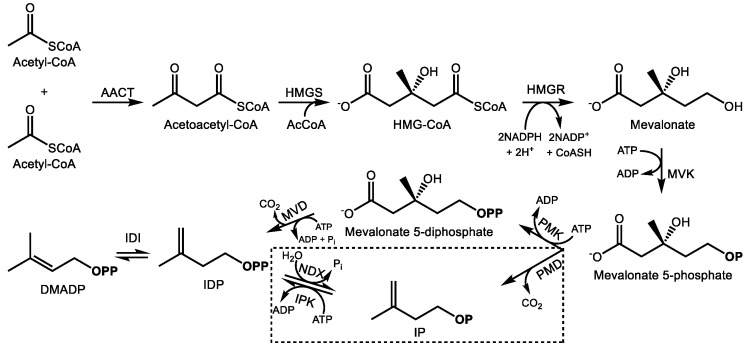
Biosynthesis of isopentenyl (IDP) and dimethylallyl diphosphate (DMADP) via the mevalonic acid (MVA) pathway and alternative MVA pathway (boxed) in the cytosol. Enzyme abbreviations are as follows; AACT—acetoactl-CoA thiolase, HMGS—hydroxymethylglutaryl-CoA synthase, HMGR—hydroxymethylglutaryl-CoA reductase, MVK—mevalonate kinase, PMK—phosphomevalonate kinase, MVD—mevalonate 5-phosphate decarboxylase, IDI—isopentenyl diphosphate isomerase, PMD—phosphomevalonate decarboxylase, IPK–isopentenyl phosphate kinase, NDX—Nudix hydrolase, AcCoA—acetyl-CoA. **OP** and **OPP** signify mono- and diphosphate moieties, respectively. P_i_ represents inorganic phosphate.

**Figure 2:**
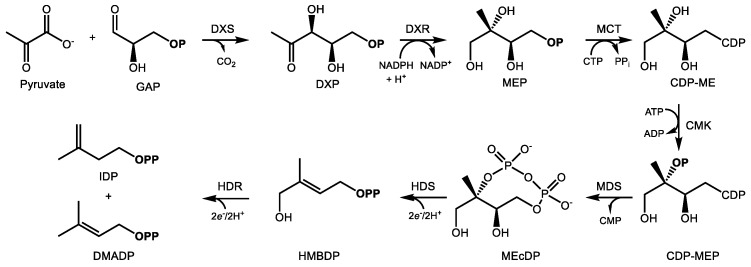
Biosynthesis of IDP and DMADP via the MEP pathway in the plastid. Enzyme abbreviations are as follows: DXS—1-deoxy-d-xylulose 5-phosphate synthase, DXR—1-deoxy-d-xylulose 5-phosphate reductoisomerase, MCT—2*C*-methyl-d-erythritol 4-phosphate cytidyltransferase, CMK—4-(cytidine 5′-diphospho)-2*C*-methyl-d-erythritol kinase, MDS—2*C*-methyl-d-erythritol-2,4- cyclodiphosphate synthase, HDS—4-hydroxy-3-methylbut-2-enyl diphosphate synthase, HDR—4-hydroxy-3-methylbut-2-enyl diphosphate reductase. For intermediates, GAP—d-glyceraldehyde- 3-phosphate, DXP—1-deoxy-d-xylulose 5-phosphate, MEP—2*C*-methyl-d-erythritol 4-phosphate, CDP–cytidyl diphosphate, MEcDP—2*C*-methyl-d-erythritol-2,4-cyclodiphosphate, HMBDP—1-hydroxy-2-methyl-2-(*E*)-butenyl-4-diphosphate, IDP—isopentenyl diphosphate, DMADP—dimethylallyl diphosphate; **OP** and **OPP** signify mono- and diphosphate groups, respectively.

The carbon backbone of highly functionalized terpenoid natural products is formed through the condensation of the central metabolic intermediates of terpenoid metabolism, isopentenyl and dimethylallyl diphosphate (IDP and DMADP) (Figure 1 and Figure 2). Two distinct biochemical pathways in nature synthesize them: the 2*C*-methyl-d-erythritol-4-phosphate (MEP) pathway and the mevalonic acid (MVA) pathway [18,19,20]. The cytosolic MVA pathway supplies IDP and DMADP in essentially all eukaryotes and the archaea, whereas the MEP pathway is functional in eubacteria and in the plastids of algae, higher plants, and protists [21]; photosynthetic organisms utilize both precursor pathways but separate them into different subcellular compartments. In plants, there is limited evidence of exchange of IDP/DMADP pools between the plastid and cytosol, usually in the context of secondary metabolism [22,23,24,25,26]. In general, the MEP pathway supplies C_5_ prenyl diphosphates for the synthesis of C_10_ monoterpenes, C_20_ diterpenes, and C_40_ tetraterpenes while the MVA pathway provides the same universal precursors for the synthesis of C_15_ sesquiterpenes, C_27–29_ sterols, C_30_ triterpenes, and their saponin derivatives.

### 2.1. The Mevalonate Pathway

The cytosolic MVA pathway produces one molecule of IDP in six enzymatic steps with the consumption of 3 acetyl-CoAs, 3ATPs, and 2 NADPH reducing equivalents [27,28] (Figure 1). The pathway begins with the Claisen condensation of two acetyl-CoA units to form acetoacetyl-CoA, a reaction carried out by acetoacetyl-CoA thiolase [29], followed by condensation with a third acetyl-CoA unit by 3-hydroxy-3-methylglutaryl-CoA synthase (HMGS) to yield HMG-CoA [30]. HMG-CoA reductase (HMGR) then reduces HMG-CoA to mevalonate at the endoplasmic reticulum (ER) surface [31], the main regulatory step of the MVA pathway, consuming 2 NADPH molecules in the process. Mevalonate kinase then phosphorylates mevalonate into mevalonate-5-phosphate [32] which is followed by a second phosphorylation into mevalonate 5-diphosphate by phosphomevalonate kinase. The final step of the MVA pathway is the ATP-dependent decarboxylation of mevalonate diphosphate into IDP by mevalonate diphosphate decarboxylase [33]. There is increasing evidence that the final steps of the MVA pathway take place in the peroxisome [34,35]. Following IDP formation, it can be reversibly isomerized into DMADP by IDP isomerase [36] and further converted into the C_15_ prenyl diphosphate farnesyl diphosphate (FDP) by FDP synthase [37], a member of the prenyl transferase family of enzymes which condense DMADP with a variable number of IDP units to produce prenyl diphosphates, usually of 10, 15, or 20 carbons, although longer chain prenyl diphosphates are also known. FDP is the last common intermediate among most MVA-derived plant terpenoids.

These reactions are highly conserved within plants; however, an alternative MVA route that operates simultaneously was recently identified (Figure 1). It resembles the ancestral MVA pathway in archaea wherein the last two steps are reversed, i.e., mevalonate 5-phosphate is first decarboxylated to isopentenyl phosphate, which is then phosphorylated to IDP [38]. In plants, IDP and DMADP can evidently be interconverted with their monophosphates forms via NUDIX hydrolase [39] and isopentenyl monophosphate kinase activities [40]. This process is thought to play a regulatory function to control terpenoid precursor availability, but its broader biological significance is still being explored.

### 2.2. The MEP Pathway

The MEP pathway provides IDP and DMADP for terpenoid biosynthesis in the plastid. Over a sequence of seven reactions (Figure 2), D-glyceraldehyde-3-phosphate (GAP) and pyruvate undergo condensation and reduction to yield IDP and DMADP at the expense of 3 ATP and 3 NADPH equivalents [19]. 1-Deoxy-d-xylulose 5-phosphate (DXP) synthase (DXS) condenses pyruvate and GAP into DXP in the committing step, an enzyme which also controls flux through the MEP pathway [41]. Most plant genomes encode multiple DXS isoforms belonging to distinct phylogenetic groups, denoted type I and type II [42]. While type I genes are expressed in photosynthetic tissue, the type II DXS clade is frequently associated with the biosynthesis of terpenoid secondary metabolites [43,44,45]. This class of DXS may therefore represent a biotechnological target for the manipulation of specialized terpenoids of medical interest. Type II DXS genes are often preferentially expressed in tissues where secondary metabolites accumulate, such as trichomes [46,47] or roots [48,49].

Following DXP formation, DXP reductoisomerase (DXR) then reduces it to MEP through an NADPH-dependent isomerization [50]. Following activation of MEP with a cytidine diphospho linkage at C4 [51] and phosphorylation of the alcohol at C2, the enzyme 2*C*-methyl-d-erythritol-2,4-cyclodiphosphate (MEcDP) synthase (MDS) then cyclizes this activated intermediate to the unusual 8-membered phosphodiester ring, MEcDP [52,53]. MEcDP is then reduced to 1-hydroxy-2-methyl-2-(*E*)-butenyl-4-diphosphate (HMBDP) by the iron-sulfur protein HMBDP synthase (HDS) [54,55] and then reduced again by HMBDP reductase (HDR), which produces both IDP and DMADP in a ratio of about 5:1 [56,57]. IDP isomerase is also present in the plastid, presumably to maintain IDP/DMADP ratios at optimal levels [58]. In analogy with FDP synthase noted above, the principal prenyl transferases which supply plastidic terpene biosynthesis include geranyl diphosphate (GDP) and geranylgeranyl diphosphate (GGDP) synthase, which provide C_10_ and C_20_ prenyl diphosphates for the synthesis of mono- and diterpenes, respectively. In chloroplasts, the MEP pathway mainly provides precursors for the synthesis of carotenoids and phytol for photosynthesis, along with other primary metabolites [59]. Secondary metabolites, such as those with uses in medicine, are often made in specialized, pigment-free plastids known as leucoplasts located in non-photosynthetic glandular trichomes [60,61].

## 3. Medicinal Monoterpenes

Monoterpenes are C_10_, plastid derived terpenoids, often with appreciable volatility. As such, they are frequent constituents of plant essential oils. Their anti-microbial action has been attributed to their general membrane disrupting properties [62]. Below we described two class of monoterpenoids with well documented pharmacological properties, the volatile *p*-menthane (−)-menthol and the cannabinoids, whose carbon skeleton includes portions derived from GDP as well as a polyketide fragment and a fatty acid contribution of variable length.

### 3.1. (−)-Menthol and Related p-Menthanes from the Lamiaceae

**Figure 3:**
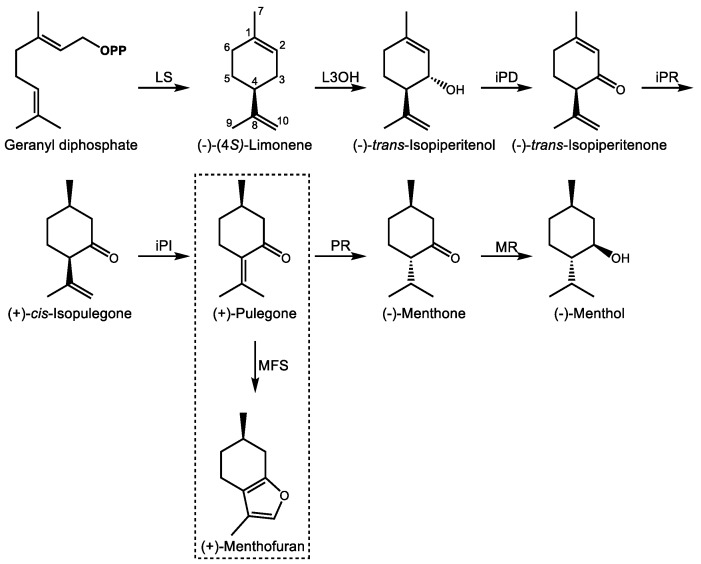
Steps in the biosynthesis of (−)-menthol and related *p*-menthanes in peppermint (*M. x piperita).* Enzyme abbreviations are as follows; LS–(−)-limonene synthase, L3OH—(−)-limonene 3-hydroxylase, iPD— (−)-*trans*-isopiperitenol dehydrogenase, iPR—(−)-*trans*-isopiperitenone reductase, iPI—(+)-*cis*-isopulegone isomerase, PR—(+)-pulegone reductase, MR—(−)-menthone reductase, MFS—(+)-menthofuran synthase. **OPP** signifies a pyrophosphate group. The box indicates the branch pathway leading to (+)-menthofuran.

The *Mentha* genus (Lamiaceae) provides several *p*-menthane monoterpenoids of pharmacological interest. (−)-Menthol (Figure 3), a major constituent of the essential oil of peppermint (*Mentha* x *piperita*), has been known since the 1950s to act as a full agonist of the Cold and Menthol Receptor 1 (CMR1) [63]. The CMR1 receptor, also known as ‘transient receptor potential melastatin 8′ (TRPM8), is a cold-sensitive, homotetrameric Na^+^/Ca^2+^ ion channel expressed in sensory neurons [64]. Its activation by cold, positive membrane potential, phosphatidylinositol-4,5-bisphosphate, or (−)-menthol produces a cold sensation, although the gating mechanism is different in each case [65,66]. This stands in contrast to other plant natural products such as capsaicin, which activates the heat sensitive TRP vanilloid 1 receptor to produce the opposite effect [67]. The Ca^2+^ blocking properties of (−)-menthol also confer mild pain relieving and analgesic effects through the indirect activation of the κ opioid receptor [68], relaxation of smooth muscle, and positive allosteric activation of the GABA_A_ receptor [69]. These pharmacological attributes may explain its traditional and current use in relieving muscle pain and in the treatment of irritable bowel syndrome [70], in addition to its better known role as a flavoring agent in oral hygiene products and as a food additive.

The biosynthesis of (−)-menthol begins with GDP (Figure 3), the immediate precursor to most monoterpenes [71] with rare exceptions [72]. GDP itself is formed through the condensation of IDP and DMADP through the action of GDP synthase (GDS), a heterodimer with a large catalytic and smaller chain length specificity subunit first cloned from peppermint (*Mentha x piperita* L.) and spearmint (*Mentha spicata* L.) [73]. (−)-Limonene synthase acts on GDP to produce the cyclic, olefinic monoterpene (−)-limonene with loss of pyrophosphate as the first committed intermediate [74], to which (−)-limonene-3-hydroxylase then introduces an alcohol function at C3 to form (−)-*trans*-isopiperitenol [75]. (−)-*trans*-Isopiperitenol dehydrogenase next oxidizes this to (−)-*trans*-isopiperitenone [76] followed by its reduction to (+)-*cis*-isopulegone [77]. (+)-*cis*-Isopulegone isomerase then yields (+)-pulegone [78], the branch-point intermediate leading alternately to (+)-menthofuran or the menthones [79]. In the formation of (−)-menthol, (+)-pulegone reductase reduces (+)-pulegone to (−)-menthone [77]. (−)-Menthol is then formed by a final reduction by (−)-menthone:(−)-menthol reductase [80]. These seven enzymatic steps begin in the plastid, move to the ER, continue in the mitochondria, and the final steps are completed in the cytosol [81]. Ultimately, the products are secreted into the subcuticular storage space of the oil gland. Developmental regulation of (−)-menthol biosynthesis in glandular trichomes of peppermint occurs primarily at the transcriptional level [82].

The metabolic control of this process has been the subject of extensive mathematical modeling for the purpose of engineering oil quality [14,83,84,85]. Since peppermint is a sterile hybrid, plant breeding is not available as a means to improve oil yields and quality, for instance, by reducing (+)-menthofuran content, an undesirable side product resulting from the oxidation of (+)-pulegone by (+)-menthofuran synthase, a cytochrome P450 monooxygenase [79]. Indeed, the toxicity of pennyroyal (*M. pulegium*) is due to its high (+)-pulegone content, which when ingested is similarly converted to (+)-menthofuran by a different P450 enzyme in the human liver [86]. The uterine contraction-inducing, hepatotoxic properties of pennyroyal are due to this bioactivation and explain the historical use of its essential oil as an abortifacient and emmenagogue in folk medicine dating to at least 421 B.C. [87], despite its obvious toxicity in concentrated form. Biotechnological approaches have successfully reduced (+)-menthofuran content in peppermint through antisense silencing of (+)-menthofuran synthase while simultaneously increasing yields of the desirable (−)-menthol through upregulation of DXR, the second enzyme of the MEP pathway [88].

### 3.2. Medicinal Monoterpene Derived Meroterpenes: Cannabinoids

‘Cannabinoid’ originally referred to the group of prenylated phenolic compounds from *Cannabis spp.* (Cannabaceae) but now includes any ligand capable of specifically binding to the human cannabioid receptors, even endogenously produced cannabinoids with no structural similarity to their plant-derived, terpenophenolic counterparts [89]. Here we focus exclusively on cannabinoids of plant origin. *C. sativa* L. and *C. indica* Lam. (marijuana) have been used for their psychoactive, anxiolytic and anesthetic effects for thousands of years [90]. Like *p*-menthane monoterpenoids in the Lamiaceae, cannabinoids accumulate in glandular trichomes, but in *Cannabis spp.*, cannabinoid-rich trichomes occur primarily in calyces and bracts of female flowers [91].

**Figure 4:**
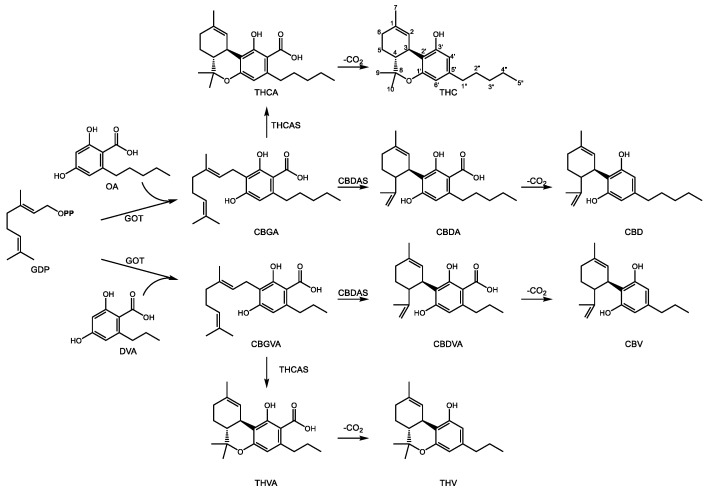
Steps in the biosynthesis of major cannabinoids in *C. sativa* and *C. indica*. Metabolites are as follows: GDP—geranyl diphosphate, OA–olivetolic acid, CBGA–cannabigerolic acid, CBDA—cannabidiolic acid, CBD—cannabidiol, THCA—Δ^9^-tetrahydrocannabinic acid, THC—Δ^9^-tetrahydrocannabinol, DVA—divarinic acid, CBGVA—cannabigerovaric acid, CBDVA—cannabidivarinic acid, CBV—cannabivarin, THVA—tetrahydrocannabivarinic acid, THV—tetrahydrocannabivarin. Enzymes are as follows: GOT—geranylpyrophosphate:olivetolate geranyl transferase, THCAS—tetrahydrocannabinolic acid synthase, CBDAS—cannabidiolic acid synthase, THCAS—tetrahydrocannabinolic acid synthase, CBDAS—cannabidiolic acid synthase. The traditional name Δ^9^-THC is used in the text while the monoterpene numbering scheme is used in this figure. **OPP** indicates a diphosphate group.

The structure of cannabinoids includes a C_10_ GDP derived monoterpene backbone, a polyketide resorcinol ring, and an acyl chain of variable length, usually 3- or 5-carbons [92]. The two best known cannabinoids from *C. sativa* include Δ^9^-tetrahydrocannabinol (THC) and cannabidiol (CBD) (Figure 4), known for their psychoactive and pain relieving properties, respectively. They share a common set of early biosynthetic steps and diverge at the central intermediate cannabigerolic acid (CBGA), a central intermediate in cannabinoid biosynthesis. Geranylpyrophosphate:olivetolate geranyl-transferase (GOT) produces CBGA through the prenylation of olivetolic acid (OA) [93]. OA is the product of the condensation of hexanoyl-CoA with 3 malonyl-CoA units whose formation requires the combined action of olivetol synthase [94] as well as olivetolic acid cyclase [95]. A gene responsible for hexanoyl-CoA formation has not yet been isolated. CBGA can then undergo oxidative cyclization to CBD acid (CBDA) or THC acid (THCA) through the action of one of two related enzymes. In the first, CBDA synthase, a flavin-bearing enzyme, oxidatively cyclizes the geranyl group through a mechanism involving deprotonation of the terminal methyl group on the geranyl chain, leaving a free propylene group [96]. Conversely, CBGA may also undergo a similar oxidative cyclization by THCA synthase [97]. In this case, deprotonation of the carbocationic intermediate occurs on the C1′ hydroxyl group of the alkylresorcinol ring which then attacks the propylene tail, leading to formation of a third ring [98]. This additional ring distinguishes THCA from CBDA, although the enzyme mechanisms are nearly identical. Both CBDA and THCA then undergo spontaneous decarboxylation to form the biologically active components CBD and THC, respectively [92].

CBDA and THCA bear a characteristic pentyl side chain on C5′ of the resorcinolic acid ring. However, other pharmacologically active cannabinoids containing acyl groups of different lengths [99] are also known. Propyl substituted analogs of CBDA and THCA exist in the form of cannabidivarinic acid (CBDVA) and tetrahydrocannabivarinic acid (THCVA) (Figure 4). It is likely they are formed by the same enzymes yielding their bi- and tri-cyclic pentyl counterparts (CBDAS and THCAS, respectively), although this area continues to be investigated. The resorcinolic acid component of propyl cannabinoids is divarinolic acid in place of OA [100], and butanoyl-CoA is the presumed acyl thioester contributing the alkyl group [101].

The principal target of THC is the cannabinoid 1 (CB1) receptor, the primary cannabinoid receptor in the central nervous system [102] whose discovery followed from the affinity of the plant compound towards CB1 [103]. This G-protein coupled receptor functions as part of an inhibitory pathway to block calcium influx in the pre-synaptic nerve needed for release of excitatory neurotransmitters such as glutamate into the synaptic cleft [104]. The targets of CBD are less clear [105,106] but both have demonstrated efficacy towards treating epilepsy [107].

**Figure 5:**
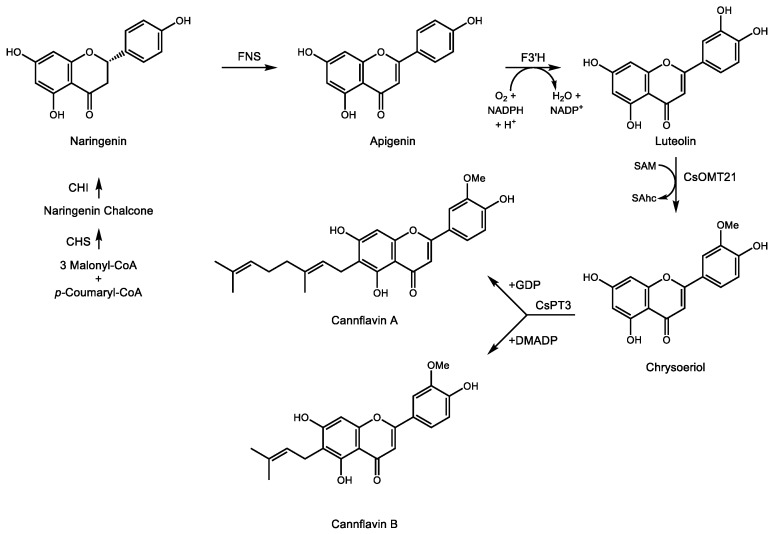
Biosynthetic pathway for the formation of cannflavin A and B in *C. sativa* and *C. indica*. This terpenophenolic compound is produced from geranyl diphosphate (GDP) or dimethylallyl diphosphate (DMADP) and the flavonoid chrysoeriol derived from naringenin in *C. sativa* and *C. indica*. SAM—S-adenosylmethionine, SAhc—S-adensoylhomocysteine. Enzymes are as follows: CHS—chalcone synthase, CHI—chalcone isomerase, FNS—flavone synthase, F3’H—flavanoid 3’ hydroxylase, CsOMT21—*O-*methyltransferase 21, CsPT3—prenyltransferase 3.

Pharmacologically active terpenophenolics from *Cannabis spp.* are not limited to cannabinoids *sensu stricto*. Two prenylated flavones, cannflavin A and B [108], result from the transfer of either a geranyl or dimethylallyl group, respectively, to the A ring of the methylated flavone chrysoeriol (Figure 5). The position of the alcohol groups on the aromatic ring relative to the site of prenylation is identical to that of the resorcinol ring of OA during the formation of CBGA. Rea et al. recently described two cDNAs involved in the formation of cannflavins [109], namely an *O*-methyl transferase converting luteolin to chrysoeriol and an aromatic prenyltransferase accepting either GDP or DMADP as prenyl donor to produce cannflavin A and B, respectively. Their analgesic and anti-inflammatory activity [110] derive from inhibition of two enzymes in the E2 prostaglandin inflammatory pathway [111]. The recent isolation of the cDNAs for the cannflavin pathway will enable a more detailed evaluation of their clinical potential for pain relief and treatment of chronic inflammation.

## 4. Medicinal Sesquiterpenes

Sesquiterpene biosynthesis normally begins in the cytosol via the C_15_ prenyl diphosphate FDP, which itself is derived from mevalonic acid and the MVA pathway. Sesquiterpene synthases act on FDP to yield hundreds of sesquiterpene hydrocarbons and alcohols based on dozens of carbon skeletons. They are frequently responsible for familiar scents and flavors, for instance ginger (gingerol), clove, cannabis, rosemary (β-caryophyllene), patchouli (patchoulol), sandalwood (α-santalene), and rain (geosmin, a bacterial sesquiterpene). They are the heaviest of the volatile terpenes under standard conditions (diterpene hydrocarbons ordinarily require heating to form gases). Plant families known to be principal producers of sesquiterpene volatiles include the Lamiaceae, Geraniaceae, Rutaceae, Myrtaceae, Cannabaceae, and Gingeraceae. The use of these essential oils in traditional herbal medicine such as aromatherapy and Ayurvedic medicine is well documented [112]. There is currently little evidence their use confers actual medical benefits, yet their use remains widespread due to their aesthetic, historical, and cultural significance. On the other hand, a number of highly functionalized, non-volatile sesquiterpenes of plant origin have demonstrated highly specific biological activity and have therefore been investigated for their clinical potential. A selection of these medically useful plant sesquiterpenoids follows.

### 4.1. Artemisinin

Wormwood (*Artemisia annua* L., or qinghaosu, Asteraceae) is native to China and is the source of the sesquiterpene endoperoxide artemisinin, shown to be highly effective at treating malaria [113,114]. In the early 1970s, phytochemists such as Youyou Tu investigated plants used in traditional Chinese herbal medicine and identified the usefulness of the traditional remedy qinghaosu in eradicating *Plasmodium spp*. infections, the causative agent of malaria, for which she received a Nobel Prize in Medicine in 2015 [115]. It is more effective against a broader range of life cycle stages of the apicomplexan parasite than traditional anti-malarials such as quinine [116].

**Figure 6:**
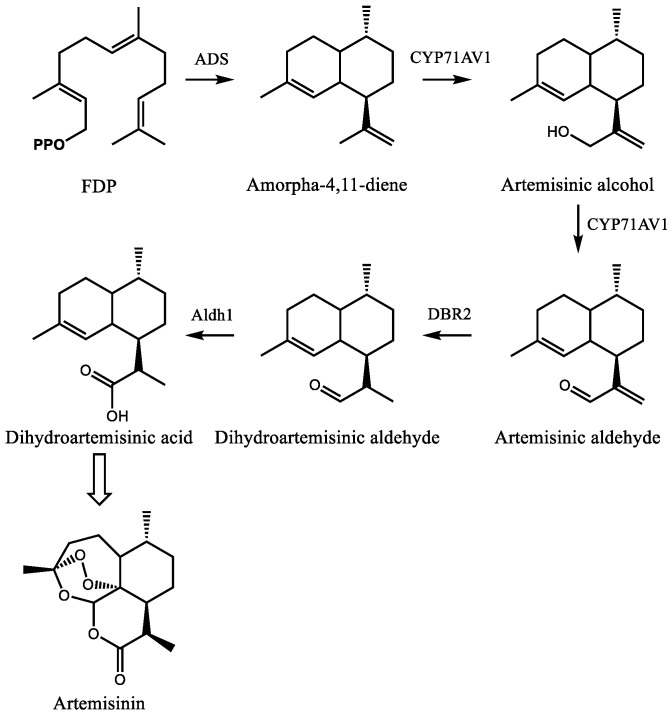
Steps in the biosynthesis of the sesquiterpene lactone artemisinin in *A. annua*. Enzyme abbreviations are as follows: ADS–amorpha-4,11-diene, CYP71AV1–cytochrome P450 71AV1, DBR2–double bond reductase 2, Aldh1–aldehyde dehydrogenase 1. **OPP** signifies a diphosphate moiety and FDP represents farnesyl diphosphate. The open arrow indicates photooxidative steps involved in the formation of the endoperoxide ring which remain uncharacterized.

Artemisinin is a sesquiterpene lactone with an unusual endoperoxide ring which imparts its anti-malarial properties (Figure 6). The mechanism of action is still unclear [117], and several possibilities have been proposed [118,119,120]. Most involve the degradation of the endoperoxide bridge in a heme-dependent process to form carbon centered radicals which then alkylate multiple targets including heme and proteins. The involvement of heme is consistent with the specificity of artemisinin towards *Plasmodium spp*. [116]. The direct cause of toxicity to the parasite is unclear but may involve interference with the conversion of heme to hemozoin [121], although this hypothesis has been disputed [122] (and reviewed in reference [123]).

The early stages of artemisinin biosynthesis in glandular trichomes of *A. annua* are now understood at the biochemical and molecular levels, although the exact mechanism of the later stages remains unclear. Current efforts now focus on the regulation of this pathway in an effort to improve artemisinin production directly in plants. The pathway begins with the conversion of FDP to amorpha-4,11-diene by amorpha-4,11-diene synthase (Figure 6) [124,125]. Biochemical characterization and metabolite profiling of plant tissue indicated the presence of this olefin as well as its oxygenated derivatives artemisinic alcohol and aldehyde, dihydroartemisinic alcohol and aldehyde, and dihydroartemisinic acid [126]. Following the cloning and expression of CYP71AV1, which converts amorpha-4,11-diene to artemisinic alcohol and aldehyde [127], Zhang et al. characterized the artemisinic aldehyde Δ11(13) reductase (Double bond reductase 2, Dbr2), which preferentially reduces artemisinic aldehyde to dihydroartemisinic aldehyde [128]. This substrate is then converted to dihydroartemisinic acid by aldehyde dehydrogenase 1 (Aldh1) [129]. From dihydroartemisinic acid, the remaining steps involve the photooxidative formation of the endoperoxide ring. However, the details of this process, such as the potential involvement of additional enzyme activities, are currently unclear.

### 4.2. Thapsigargin

Thapsigargin is a highly functionalized 6,12-guianolide sesquiterpene lactone produced by *Thapsia garganica* (Apiaceae), a perennial shrub native to the western Mediterranean basin. Its resin elicits contact dermatitis [130]. As such, it is sometimes referred to as ‘deadly carrot’. However, it has long been utilized in European and North African herbal medicine traditions for rheumatism, sterility, and colds [131] and its use in Algeria for circulatory illnesses dates to the 19th century [132]. Patkar, et al. reported its histamine releasing properties in the late 1970s [133], and its protein target in mammalian cells was later identified as the sarcoplasmic reticulum Ca^2+/^ATPase pump (SERCA) [134]. Following additional characterization of its properties as a SERCA inhibitor [135], its apoptosis inducing potential became apparent [136]. SERCA is responsible for pumping cytosolic Ca^2+^ into the sarcoplasmic reticulum in myocytes during muscle contraction/relaxation cycles. Thapsigargin mediated inhibition of SERCA therefore blocks muscle relaxation by preventing removal of Ca^2+^ ions from the cytosol. Thapsigargin induces endoplasmic reticulum stress generally, and one consequence of this is the inhibition of autophagosome fusion with the lysosome during autophagy, resulting in apoptosis [137]. Thus, its ability to disrupt Ca^2+^ homeostasis in mammalian cells suggested a potential role in cancer therapy, yet its high cytotoxicity and lack of specificity for tumor cells precluded further development as an anti-cancer drug until recently. Denmeade et al. successfully coupled thapsigargin to a prostate specific antigen (PSA) [138], effectively generating a prostate cancer targeted prodrug. Mipsigargin, a thapsigargin-based variant of this PSA-coupling approach targeting solid tumors [139], is now in phase II clinical trials for hepatocellular carcinoma [140]. Yields of thapsigargin are low from *T. garganica* (<0.5% by dry weight), and the plant is difficult to cultivate, making a detailed characterization of the relevant genes and enzymes instrumental to achieving industrial scale production.

**Figure 7:**
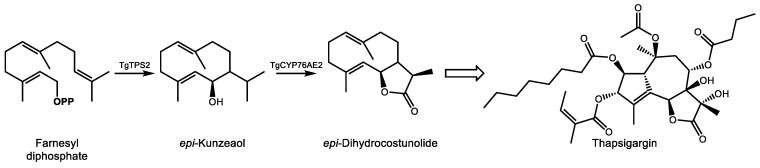
Initial biosynthetic steps for the formation of the sesquiterpene lactone thapsigargin in *T. garganica*. Enzyme abbreviations are as follows: TgTPS2–*epi*-kunzeaol synthase, TgCYP76AE2–cytochrome P450 76AE2. **OPP** signifies a diphosphate moiety. The open arrow indicates uncharacterized steps between *epi*-dihydrocostunolide and thapsigargin.

Our current knowledge of the biosynthesis of thapsigargin is limited, and complete cDNA cloning and biochemical characterization have been accomplished for only the initial steps to date (reviewed in [141]). In the committing step of the pathway, the sesquiterpene synthase TgTPS2 converts FDP into the sesquiterpene alcohol *epi*-kunzeaol (Figure 7) [142]. Screening of *T. garganica* cytochrome P450s by transient expression in *Nicotiana benthamiana* followed by mass spectrometry analysis demonstrated that only one enzyme, TgCYP76AE2, was capable of transforming *epi*-kunzeaol into a product, identified as the germacrenolide sesquiterpenes lactone *epi*-dihydrocostunolide [143]. Unlike the biosynthesis of costunolide, which requires two separate cytochrome P450s to form the alcohol and acid groups prior to lactonization, the preexistence of a C6 hydroxyl group in *epi*-kunzeaol allows for direction lactone ring formation following a triple oxidation at C12 by TgCYP76AE2, a reaction similar to that of germacrene A oxidase [144]. Andersen et al. also reported detection of transcripts for TgTPS2 and TgCYP76AE2 in the epithelial cells lining secretory ducts of *T. garganica* root sections. Imaging mass spectrometry detection of thapsigargin in structures resembling secretory ducts corroborated these observations, strongly implicating epithelial cells in the early steps of thapsigargin biosynthesis [143]. Additional reactions to reach thapsigargin that currently remain to be elucidated include, at a minimum, closure of the 10-membered *epi*-dihydrocostunolide ring into five and seven membered rings, six additional hydroxylations, and four acylations. The difficulty in detecting additional intermediates in root extracts which might provide clues as to the predominant reaction sequence in vivo could be an indication of the involvement of a metabolon structure which efficiently transfers metabolic intermediates between active sites [145]. Improvements in bioinformatics approaches and transient expression technologies which mitigate the necessity of obtaining synthetic substrates will no doubt play a role in the elucidation of the remaining steps of the pathway.

## 5. Medicinal Diterpenoids

In addition to C_10_ monoterpenoids, the plastid is also the site of several C_20_ plant diterpenoids of medical significance. Although not all reactions in these complex pathways take place in the plastid, the initial steps leading to the prenyl diphosphate precursor of diterpenes, GGDP, and the cyclization to an olefinic hydrocarbon by a terpene synthase occur here.

### 5.1. Paclitaxel

**Figure 8:**
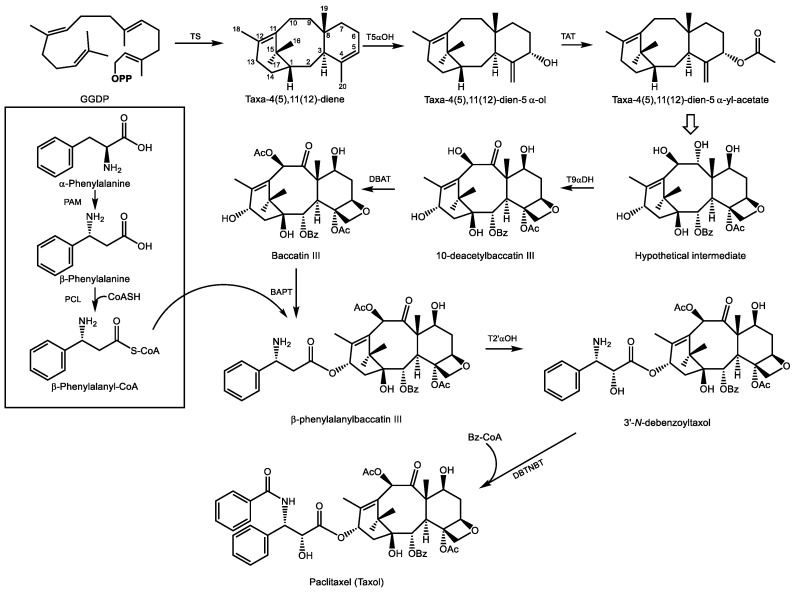
Simplified pathway for the biosynthesis of paclitaxel (taxol) in *T. brevifolia* (pacific yew). GGDP–geranylgeranyl diphosphate, **OPP**–diphosphate, Bz–benzyl, Ac–acetyl, CoA–Coenzyme A. Enzymes: TS–taxa-4(5),11(12)-diene, T5αOH–taxadiene-5α-hydroxylase, TAT–taxadiene-5α-ol-*O*-acetyl transferase, T9αDH–taxane-9α-dehydrogenase, DBAT–10-deacetylbaccatin III-10-*O*-acetyl transferase, BAPT–baccatin III:3-amino,13-phenylpropanoyl transferase, T2’-αOH–taxane-2’α-hydroxylase, DBTNBT–debenzoyltaxol *N*-benzoyl transferase, PAM–phenylalanine aminomutase, PCL–β-phenylalanyl-CoA ligase. The open arrow represents multiple oxidative steps to arrive at the hypothetical intermediate.

The noted anti-neoplastic agent paclitaxel (also known by its trademarked name taxol^®^) was first isolated from the bark of the pacific yew tree (*Taxus brevifolia*) and structurally elucidated in 1971 [146]. A substantial effort was required to elucidate its structure (Figure 8) due to the complexity of its functional groups. Paclitaxel possesses eight oxygen functional groups, including two with acetylations, a benzoylation, an oxetane ring, and a side chain attachment at C13 composed of a β-phenylalanyl group which is further modified by hydroxylation and *N*-benzoylation [147]. Evidence for its mechanism of action as a microtubule stabilizer and inhibitor of mitosis [148] emerged a few years later, but FDA approval for use in refractory ovarian cancer and metastatic breast cancer was not issued until 1992 and1994, respectively [149]. Much of this delay was due to the exceptionally low abundance of paclitaxel in plant tissue, a restriction which to this day limits the world wide availability of what is now the most commercially successful anti-cancer agent with combined annual sales of derivatives and generics approaching ten billion USD [150,151].

*Taxus* cell cultures, microbial fermentation, and semi-synthesis are now the principal avenues for producing pharmaceutical grade paclitaxel since harvesting yew bark is destructive and yields only ~1 g product from three adult trees, a slow growing and threated species which cannot meet the global demand without risk of extinction [152]. The biosynthesis *in planta* has been extensively studied, and most of the 19 enzymatic steps have been characterized. However, uncertainty regarding the order of several hydroxylations and acetylations remain. Indeed, feeding studies suggest multiple routes to paclitaxel operate in parallel in the plant, along with several metabolic dead ends resulting in taxoids with lesser or no biological activity such as (+)-taxusin, an abundant, non-bioactive taxoid which nonetheless provides a generic, readily available substrate to test the activities of candidate cytochrome P450s [153]. The order of some reactions in the natural pathway may be somewhat variable. Below, we highlight what is generally considered to be the dominant path to paclitaxel.

GGDP is generally considered the precursor to all diterpenoids. It results from GGDP synthase activity which combines one unit of DMADP with 3 units of IDP from the MEP pathway. The first committed reaction in paclitaxel biosynthesis is the cyclization of GGDP to form taxa-4(5),11(12)-diene, carried out by taxadiene synthase [154] (Figure 8). This olefin is then substrate for taxadiene-5α-hydroxylase, an ER localized cytochrome P450 which employs a radical mechanism that abstracts a H atom from the C20 methyl group, generating an allylic radical to which oxygen is added to produce taxa-4(20),11(12)-dien-5α-ol [155]. Acetylation of this alcohol in the cytosol by taxadiene-5α-ol-*O*-acetyl transferase yields taxa-4(20),11(12)-dien-5α-yl-acetate [156].

Considerable uncertainty regarding the order of the next eight steps leads to multiple possible intermediates which converge on a hypothetical intermediate (Figure 8) which now includes all eight oxygen functional groups. As shown here, this intermediate, now contains the four-membered oxetane ring which confers binding affinity to β-tubulin and is therefore critical to the biological activity of paclitaxel. Studies on the oxetane ring formation have provided two mechanistic explanations for its formation: one involves an epoxidation of the ene-acetoxy group of taxa-4(20),11(12)-dien-5α-yl-acetate to yield a 5-acetoxy-4(20)-epoxy intermediate, which then rearranges to the oxetane ring [152]. The other suggests that a downstream acetyltransferase, 10-deactylbaccatin III 10β-*O*-acetyltransferase (DBAT), may also acetylate the tertiary C4 position on the oxetane ring separately [157].

There is little doubt that following taxane-9α-dehydrogenase activity, which converts the alcohol function at C9 to a ketone group (uncharacterized but described by [150]), DBAT then converts 10-deacetylbaccatin III into baccatin III [158]. Attachment of the eventual phenylisoserine side chain at C13 follows. This begins with conversion of α-phenylalanine to β-phenylalanine, a reaction catalyzed by phenylalanine aminomutase [159], and its ligation to coenzyme A [160]. Following the transfer of this acyl group to C13 of baccatin III [161], the final steps include a 2′-hydroxylation of the phenylpropyl side chain (an as of yet uncharacterized enzyme) and *N*-benzoylation of the amino group to yield paclitaxel [162]. As of this date, a total of five cDNAs encoding biosynthetic enzymes in the paclitaxel pathway remain to be isolated and characterized. A complete cohort of biosynthetic genes for paclitaxel formation will enable improvements to sustainable cell culture and microbial production systems to ensure a regular supply of this drug without compromising threatened populations of the pacific yew tree from which it was originally isolated.

### 5.2. Ingenol Mebutate and Related Phorbol Esters

**Figure 9:**
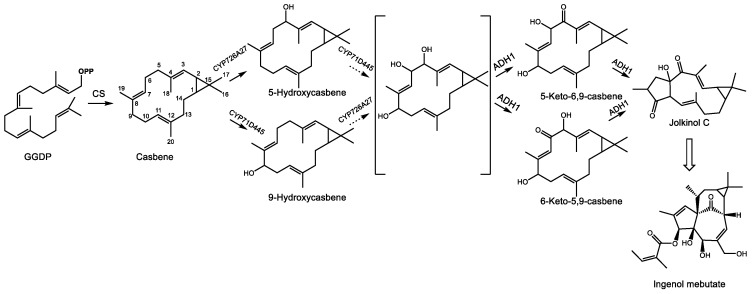
Proposed pathway for the initial steps leading to the *Euphobia* factor ingenol mebutate in *Euphorbia spp.*, as per [168]. GGDP represents geranylgeranyl diphosphate and OPP signifies a diphosphate group. Enzyme abbreviations: CS-casbene synthase, CYP726A27–cytochrome P450 726A27, CYP71D445–cytochrome P450 71D445, ADH1–alcohol dehydrogenase 1.

The family Euphorbiaceae features a wide variety of toxic and pharmacologically useful diterpenoids in the phorbol ester and ingenol subclasses. Most, if not all, of these secondary metabolites appear to be derived from the diterpene olefin casbene (Figure 9), an early prediction made upon the initial characterization of casbene synthase from *Ricinus communis* [163] that has since been corroborated by the isolation of an *R. communis* cDNA encoding casbene synthase [164] and transcriptome mining, cloning, and expression of diterpene synthases from multiple Euphorbiaceae species. With one exception, all appear to use casbene synthase as the singular diterpene synthase in their respective genomes beyond those of primary metabolism [165]. The use of a common terpene synthase product as a starting point for the chemical diversification of diterpenes in the Euphorbiaceae stands in contrast to the more common strategy for generating terpenoid diversity in plants at the terpene synthase step. Three diterpenoids from the Euphorbiaceae display significant pharmacological activity: ingenol mebutate (described further below), the latent HIV-1 activator prostratin [166], and the analgesic resiniferatoxin with properties similar to capsaicin [167].

Ingenol 3-mebutate, from *Euphorbia peplus* and *E. lathyrus*, has been approved for treatment of actinic keratosis [169] due to its ability to interact with protein kinase C (PKC) [170]. Most phorbol ester PKC-activators such as phorbol 12-myristate 13-acetate are potent mitogens [171,172]. However, ingenol 3-mebutate displays anti-tumor [173] and anti-leukemic effects [174] while also promoting PKC.

Little is known regarding the biosynthesis of ingenol 3-mebutate, however, cyclization of the C_20_ prenyl diphosphate precursor, GGDP, to casbene has been proposed as the first committed step in this pathway [165]. However, to obtain the 5/7/7/3 ring system of the polycyclic ingenane carbon skeleton (Figure 9), this macrocyclic intermediate would need to form two C-C bonds to generate the four ring ingenane system. The absence of casbene-like structures containing only 6 or 7 membered rings in this genus and the large number of lathyrane type skeletons bearing a 5-membered ring [175] have led some investigators to speculate that the 6,10 ring closure to yield a lathyrane skeleton is the next C-C bond formed en route to the ingenane system seen in ingenol mebutate [165]. Luo et al. point out that polycyclic diterpenoids display a higher degree of oxygenation than bicyclic casbene types [168], suggesting that cyclization may depend on the regiospecific addition of oxygen groups, particularly at C9, which may represent an early bifurcation step between polycyclic skeletons of the lathryane, tigliane, and ingenane classes and macrocyclic casbene type diterpenoids. Using cDNAs obtained from seeds of *E. lathyris*, they then present co-expression evidence using casbene synthase, two cytochrome P450s, and an alcohol dehydrogenase transiently expressed in tobacco which demonstrate the dehydrogenase-dependent cyclization in this pathway to form the 5-membered lathyrane jolkinol C, which naturally occurs in *E. jolkini* [176]. The authors propose the following biosynthetic steps for ingenol 3-mebutate: following the formation of casbene, two regiospecific hydroxylations carried out by CYP71D445 and CYP726A27 introduce alcohol groups at C9 and C5, respectively. One of the aforementioned cytochromes then adds a third hydroxylation at C6 to produce a triol. ADH1 then dehydrogenates the alcohol function at C5 or C6, leading to the formation of the C6-C10 bond, possibly through an enol-tautomer mechanism. Additional steps leading from jolkinol C to ingenol 3-mebutate are presently unknown, but efforts to elucidate the remaining steps of this pathway using transient expression in tobacco as a screening system as well as traditional biochemical characterization are currently underway.

## 6. Conclusions

The evolution of highly functionalized plant terpenes was punctuated by rapid proliferation of terpene synthase and cytochrome P450 gene families [6], resulting in chemical diversification across the plant kingdom through the introduction of oxygen functional groups into the olefinic backbone of terpene hydrocarbons (for example, see (−)-menthol–Section 3.1 and artemisinin–Section 4.1) and condensations with derivatives from other natural products families, such as short and branched chain fatty acids (cannabinoids–Section 3.2, ingenol mebutate–Section 5.2, and thapsigargin–Section 4.2), amino acids (paclitaxel–Section 5.2), and phenolic compounds (cannabinoids and cannflavin A and B–Section 3.2). Both the cytosolic MVA pathway and the plastid localized MEP pathway contribute terpene skeletons leading to functionalized and biologically active secondary metabolites. While some plant terpenes such as β-carotene, phylloquinone and tocopherols constitute essential (pro)vitamins in the human diet, others, such as phytosterols and essential oils, provide nutritional benefits or act as beneficial anti-oxidants. Here, however, we have focused on compounds with highly specific pharmacological activities. We have also endeavored to highlight lesser known plant terpenes whose clinical evaluation has been hampered by poor availability and intractable biochemistry, but whose clinical potential could be facilitated by elucidation of the corresponding biosynthetic pathways in coming years.

A major question surrounding plant terpenes of medical relevance concerns why this group of plant natural products yields clinically valuable compounds at such a high rate compared to unbiased combinatorial synthesis. Since plants are sessile organisms, their survival depends partly on the ability of specialized metabolites to manipulate animals in their environment for pollination and defense, and some of these natural products fortuitously exhibit properties useful in medicine although this is uncommon. It is likely that the medically useful properties of plant terpenes are exaptive, and largely a reflection of their chemical ecological roles as plant defense compounds against insect herbivores [4,177]. The emergence of highly potent and medically useful plant terpenes may therefore exploit natural similarities between human and insect physiology as well as a higher compatibility with proteins compared to synthetic compounds. As defense compounds, high potency and specificity are essential for effectiveness as feeding deterrents, particularly in light of the high metabolic cost of producing and storing such secondary metabolites [178]. Future efforts aimed both at optimizing screening strategies for plant terpenoid lead compounds as well as new approaches to elucidate complex biochemical pathways of existing candidates will undoubtedly improve the discovery rate and availability of plant terpenoids for use in medicine.

## References

[1] Bouvier F., Rahier A., Camara B. (2005). Biogenesis, molecular regulation and function of plant isoprenoids. Prog. Lipid Res..

[2] Rasmann S., Kollner T.G., Degenhardt J., Hiltpold I., Toepfer S., Kuhlmann U., Gershenzon J., Turlings T.C.J. (2005). Recruitment of entomopathogenic nematodes by insect-damaged maize roots. Nature.

[3] McCormick A.C., Unsicker S.B., Gershenzon J. (2012). The specificity of herbivore-induced plant volatiles in attracting herbivore enemies. Trends Plant. Sci..

[4] Gershenzon J., Dudareva N. (2007). The function of terpene natural products in the natural world. Nat. Chem. Biol..

[5] Weng J.-K., Philippe R.N., Noel J.P. (2012). The rise of chemodiversity in plants. Science.

[6] Boutanaev A.M., Moses T., Zi J., Nelson D.R., Mugford S.T., Peters R.J., Osbourn A. (2015). Investigation of terpene diversification across multiple sequenced plant genomes. Proc. Natl. Acad. Sci. USA.

[7] Bohlmann J., Meyer-Gauen G., Croteau R. (1998). Plant terpenoid synthases: Molecular biology and phylogenetic analysis. Proc. Natl. Acad. Sci. USA.

[8] Kennedy D.O., Wightman E.L. (2011). Herbal extracts and phytochemicals: Plant secondary metabolites and the enhancement of human brain function. Adv. Nutr..

[9] Wink M. (2015). Modes of action of herbal medicines and plant secondary metabolites. Medicines.

[10] Salminen A., Lehtonen M., Suuronen T., Kaarniranta K., Huuskonen J. (2008). Terpenoids: Natural inhibitors of NF-κB signaling with anti-inflammatory and anticancer potential. Cell. Mol. Life Sci..

[11] Zhao L., Chang W.-C., Xiao Y., Liu H.-W., Liu P. (2013). Methylerythritol phosphate pathway of isoprenoid biosynthesis. Annu. Rev. Biochem.

[12] Lange B.M. (2016). Online resources for gene discovery and biochemical research with aromatic and medicinal plants. Phytochem. Rev..

[13] Zi J., Mafu S., Peters R.J. (2014). To gibberellins and beyond! Surveying the evolution of (di) terpenoid metabolism. Annu. Rev. Plant Biol..

[14] Lange B.M., Mahmoud S.S., Wildung M.R., Turner G.W., Davis E.M., Lange I., Baker R.C., Boydston R.A., Croteau R.B. (2011). Improving peppermint essential oil yield and composition by metabolic engineering. Proc. Natl. Acad. Sci. USA.

[15] Kirby J., Keasling J.D. (2009). Biosynthesis of plant isoprenoids: Perspectives for microbial engineering. Annu. Rev. Plant Biol..

[16] Agrawal A.A., Petschenka G., Bingham R.A., Weber M.G., Rasmann S. (2012). Toxic cardenolides: Chemical ecology and coevolution of specialized plant–herbivore interactions. N. Phytol..

[17] Kreis W., Müller-Uri F. (2018). Biochemistry of sterols, cardiac glycosides, brassinosteroids, phytoecdysteroids and steroid saponins. Annu. Plant Rev. Online.

[18] Banerjee A., Sharkey T.D. (2014). Methylerythritol 4-phosphate (MEP) pathway metabolic regulation. Nat. Prod. Rep..

[19] Frank A., Groll M. (2017). The methylerythritol phosphate pathway to isoprenoids. Chem. Rev..

[20] Phillips M.A., León P., Boronat A., Rodríguez-Concepción M. (2008). The plastidial MEP pathway: Unified nomenclature and resources. Trends Plant. Sci..

[21] Lombard J., Moreira D. (2010). Origins and early evolution of the mevalonate pathway of isoprenoid biosynthesis in the three domains of life. Mol. Biol. Evolut..

[22] Dudareva N., Andersson S., Orlova I., Gatto N., Reichelt M., Rhodes D., Boland W., Gershenzon J. (2005). The nonmevalonate pathway supports both monoterpene and sesquiterpene formation in snapdragon flowers. Proc. Natl. Acad. Sci. USA.

[23] Hemmerlin A., Hoeffler J.F., Meyer O., Tritsch D., Kagan I.A., Grosdemange-Billiard C., Rohmer M., Bach T.J. (2003). Cross-talk between the cytosolic mevalonate and the plastidial methylerythritol phosphate pathways in Tobacco Bright Yellow-2 cells. J. Biol. Chem..

[24] Opitz S., Nes W.D., Gershenzon J. (2014). Both methylerythritol phosphate and mevalonate pathways contribute to biosynthesis of each of the major isoprenoid classes in young cotton seedlings. Phytochemistry.

[25] Adam K.P., Thiel R., Zapp J. (1999). Incorporation of 1- 1-C-13 deoxy-D-xylulose in chamomile sesquiterpenes. Arch. Biochem. Biophys..

[26] Laule O., Furholz A., Chang H.S., Zhu T., Wang X., Heifetz P.B., Gruissem W., Lange B.M. (2003). Crosstalk between cytosolic and plastidial pathways of isoprenoid biosynthesis in Arabidopsis thaliana. Proc. Natl. Acad. Sci. USA.

[27] Bach T.J., Boronat A., Campos N., Ferrer A., Vollack K.-U. (1999). Mevalonate biosynthesis in plants. Crit. Rev. Biochem. Mol. Biol..

[28] Newman J.D., Chappell J. (1999). Isoprenoid biosynthesis in plants: Carbon partitioning within the cytoplasmic pathway. Crit. Rev. Biochem. Mol. Biol..

[29] Ahumada I., Cairó A., Hemmerlin A., González V., Pateraki I., Bach T.J., Rodríguez-Concepción M., Campos N., Boronat A. (2008). Characterisation of the gene family encoding acetoacetyl-CoA thiolase in Arabidopsis. Funct. Plant Biol..

[30] Liao P., Wang H., Hemmerlin A., Nagegowda D.A., Bach T.J., Wang M., Chye M.-L. (2014). Past achievements, current status and future perspectives of studies on 3-hydroxy-3-methylglutaryl-CoA synthase (HMGS) in the mevalonate (MVA) pathway. Plant Cell Rep..

[31] Leivar P., Gonzalez V.M., Castel S., Trelease R.N., Lopez-Iglesias C., Arro M., Boronat A., Campos N., Ferrer A., Fernandez-Busquets X. (2005). Subcellular localization of Arabidopsis 3-hydroxy-3-methylglutaryl-coenzyme A reductase. Plant Phys..

[32] Lluch M.A., Masferrer A., Arró M., Boronat A., Ferrer A. (2000). Molecular cloning and expression analysis of the mevalonate kinase gene from Arabidopsis thaliana. Plant Mol. Biol..

[33] Cordier H., Karst F., Bergès T. (1999). Heterologous expression in Saccharomyces cerevisiae of an Arabidopsis thaliana cDNA encoding mevalonate diphosphate decarboxylase. Plant Mol. Biol..

[34] Simkin A.J., Guirimand G., Papon N., Courdavault V., Thabet I., Ginis O., Bouzid S., Giglioli-Guivarc’h N., Clastre M. (2011). Peroxisomal localisation of the final steps of the mevalonic acid pathway in planta. Planta.

[35] Sapir-Mir M., Mett A., Belausov E., Tal-Meshulam S., Frydman A., Gidoni D., Eyal Y. (2008). Peroxisomal localization of Arabidopsis isopentenyl diphosphate isomerases suggests that part of the plant isoprenoid mevalonic acid pathway is compartmentalized to peroxisomes. Plant Phys..

[36] Campbell M., Hahn F.M., Poulter C.D., Leustek T. (1997). Analysis of the isopentenyl diphosphate isomerase gene family from Arabidopsis thaliana. Plant Mol. Biol..

[37] Cunillera N., Arró M., Delourme D., Karst F., Boronat A., Ferrer A. (1996). Arabidopsis thaliana contains two differentially expressed farnesyl-diphosphate synthase genes. J. Biol. Chem..

[38] Dellas N., Thomas S.T., Manning G., Noel J.P. (2013). Discovery of a metabolic alternative to the classical mevalonate pathway. eLife.

[39] Henry L.K., Thomas S.T., Widhalm J.R., Lynch J.H., Davis T.C., Kessler S.A., Bohlmann J., Noel J.P., Dudareva N. (2018). Contribution of isopentenyl phosphate to plant terpenoid metabolism. Nat. Plants.

[40] Henry L.K., Gutensohn M., Thomas S.T., Noel J.P., Dudareva N. (2015). Orthologs of the archaeal isopentenyl phosphate kinase regulate terpenoid production in plants. Proc. Natl. Acad. Sci. USA.

[41] Wright L.P., Rohwer J.M., Ghirardo A., Hammerbacher A., Ortiz-Alcaide M., Raguschke B., Schnitzler J.-P., Gershenzon J., Phillips M.A. (2014). Deoxyxylulose 5-phosphate synthase controls flux through the methylerythritol 4-phosphate pathway in Arabidopsis. Plant Physiol..

[42] Walter M.H., Hans J., Strack D. (2002). Two distantly related genes encoding 1-deoxy-d-xylulose 5-phosphate synthases: Differential regulation in shoots and apocarotenoid-accumulating mycorrhizal roots. Plant J..

[43] Phillips M.A., Walter M.H., Ralph S., Dabrowska P., Luck K., Urós E.M., Boland W., Strack D., Rodríguez-Concepción M., Bohlmann J. (2007). Functional identification and differential expression of 1-deoxy-D-xylulose 5-phosphate synthase in induced terpenoid resin formation of Norway spruce (Picea abies). Plant Mol. Biol..

[44] Saladié M., Wright L.P., Garcias Mas J., Rodriguez-Concepcion M., Phillips M.A. (2014). Small gene families encode the main rate-determining enzymes for plastidial isoprenoid biosynthesis in melon. J. Exp. Bot..

[45] Kim S.M., Kuzuyama T., Chang Y.J., Song K.S., Kim S.U. (2006). Identification of class 2 1-deoxy-D-xylulose 5-phosphate synthase and 1-deoxy-D-xylulose 5-phosphate reductoisomerase genes from Ginkgo biloba and their transcription in embryo culture with respect to ginkgolide biosynthesis. Planta Med..

[46] Lois L.M., Rodríguez-Concepción M., Gallego F., Campos N., Boronat A. (2000). Carotenoid biosynthesis during tomato fruit development: Regulatory role of 1-deoxy-D-xylulose 5-phosphate synthase. Plant J..

[47] Paetzold H., Garms S., Bartram S., Wieczorek J., Urós-Gracia E.-M., Rodríguez-Concepción M., Boland W., Strack D., Hause B., Walter M.H. (2010). The isogene 1-deoxy-D-xylulose 5-phosphate synthase 2 controls isoprenoid profiles, precursor pathway allocation, and density of tomato trichomes. Mol. Plant.

[48] Yang L., Ding G., Lin H., Cheng H., Kong Y., Wei Y., Fang X., Liu R., Wang L., Chen X. (2013). Transcriptome analysis of medicinal plant Salvia miltiorrhiza and identification of genes related to tanshinone biosynthesis. PLoS ONE.

[49] Floß D.S., Hause B., Lange P.R., Küster H., Strack D., Walter M.H. (2008). Knock-down of the MEP pathway isogene 1-deoxy-D-xylulose 5-phosphate synthase 2 inhibits formation of arbuscular mycorrhiza-induced apocarotenoids and abolishes normal expression of mycorrhiza-specific plant marker genes. Plant J..

[50] Kuzuyama T., Takahashi S., Watanabe H., Seto H. (1998). Direct formation of 2-C-methyl-D-erythritol 4-phosphate from 1-deoxy-D-xylulose 5-phosphate by 1-deoxy-D-xylulose 5-phosphate reductoisomerase, a new enzyme in the non-mevalonate pathway to isopentenyl diphosphate. Tetrahedron Lett..

[51] Kuzuyama T., Takagi M., Kaneda K., Dairi T., Seto H. (2000). Formation of 4-(cytidine 5’-diphospho)-2-C-methyl-D-erythritol from 2-C-methyl-D-erythritol 4-phosphate by 2-C-methyl-D-erythritol 4-phosphate cytidylyltransferase, a new enzyme in the nonmevalonate pathway. Tetrahedron Lett..

[52] Calisto B.M., Perez-Gil J., Bergua M., Querol-Audi J., Fita I., Imperial S. (2007). Biosynthesis of isoprenoids in plants: Structure of the 2C-methyl-D-erithrytol 2, 4-cyclodiphosphate synthase from Arabidopsis thaliana. Comparison with the bacterial enzymes. Protein Sci..

[53] Richard S.B., Ferrer J.-L., Bowman M.E., Lillo A.M., Tetzlaff C.N., Cane D.E., Noel J.P. (2002). Structure and mechanism of 2-C-methyl-D-erythritol 2, 4-cyclodiphosphate synthase an enzyme in the mevalonate-independent isoprenoid biosynthetic pathway. J. Biol. Chem..

[54] Seemann M., Bui B.T.S., Wolff M., Miginlac-Maslow M., Rohmer M. (2006). Isoprenoid biosynthesis in plant chloroplasts via the MEP pathway: Direct thylakoid/ferredoxin-dependent photoreduction of GcpE/IspG. FEBS Lett..

[55] Seemann M., Wegner P., Schunemann V., Bui B.T.S., Wolff M., Marquet A., Trautwein A.X., Rohmer M. (2005). Isoprenoid biosynthesis in chloroplasts via the methylerythritol phosphate pathway: The (E)-4-hydroxy-3-methylbut-2-enyl diphosphate synthase (GcpE) from Arabidopsis thaliana is a 4Fe-4S protein. J. Biol. Inorg. Chem..

[56] Altincicek B., Kollas A., Eberl M., Wiesner J., Sanderbrand S., Hintz M., Beck E., Jomaa H. (2001). LytB, a novel gene of the 2-C-methyl-D-erythritol 4-phosphate pathway of isoprenoid biosynthesis in Escherichia coli. FEBS Lett..

[57] Botella-Pavía P., Besumbes O., Phillips M.A., Carretero-Paulet L., Boronat A., Rodríguez-Concepción M. (2004). Regulation of carotenoid biosynthesis in plants: Evidence for a key role of hydroxymethylbutenyl diphosphate reductase in controlling the supply of plastidial isoprenoid precursors. Plant J..

[58] Phillips M.A., D’Auria J.C., Gershenzon J., Pichersky E. (2008). The Arabidopsis thaliana type I isopentenyl diphosphate isomerases are targeted to multiple subcellular compartments and have overlapping functions in isoprenoid biosynthesis. Plant Cell.

[59] Ruiz-Sola M.A., Rodriguez-Concepcion M. (2012). Carotenoid biosynthesis in Arabidopsis: A colorful pathway. Arabidopsis Book.

[60] Lange B.M., Turner G.W. (2013). Terpenoid biosynthesis in trichomes—Current status and future opportunities. Plant Biotechnol. J..

[61] Lange B.M., Wildung M.R., Stauber E.J., Sanchez C., Pouchnik D., Croteau R. (2000). Probing essential oil biosynthesis and secretion by functional evaluation of expressed sequence tags from mint glandular trichomes. Proc Natl. Acad. Sci. USA.

[62] Bakkali F., Averbeck S., Averbeck D., Idaomar M. (2008). Biological effects of essential oils—A review. Food Chem. Toxicol..

[63] Hensel H., Zotterman Y. (1951). The effect of menthol on the thermoreceptors. Acta Physiol. Scand..

[64] Yin Y., Wu M., Zubcevic L., Borschel W.F., Lander G.C., Lee S.-Y. (2018). Structure of the cold-and menthol-sensing ion channel TRPM8. Science.

[65] Bandell M., Dubin A.E., Petrus M.J., Orth A., Mathur J., Hwang S.W., Patapoutian A. (2006). High-throughput random mutagenesis screen reveals TRPM8 residues specifically required for activation by menthol. Nat. Neurosci..

[66] Voets T., Owsianik G., Janssens A., Talavera K., Nilius B. (2007). TRPM8 voltage sensor mutants reveal a mechanism for integrating thermal and chemical stimuli. Nat. Chem. Biol..

[67] Caterina M.J., Schumacher M.A., Tominaga M., Rosen T.A., Levine J.D., Julius D. (1997). The capsaicin receptor: a heat-activated ion channel in the pain pathway. Nature.

[68] Galeotti N., Mannelli L.D.C., Mazzanti G., Bartolini A., Ghelardini C. (2002). Menthol: A natural analgesic compound. Neurosci. Lett..

[69] Lau B.K., Karim S., Goodchild A.K., Vaughan C.W., Drew G.M. (2014). Menthol enhances phasic and tonic GABA_A_ receptor-mediated currents in midbrain periaqueductal grey neurons. Br. J. Pharmacol..

[70] Simren M., Tack J. (2018). New treatments and therapeutic targets for IBS and other functional bowel disorders. Nat. Rev. Gastroenterol. Hepatol..

[71] Croteau R. (1987). Biosynthesis and Catabolism of Monoterpenoids. Chem. Rev..

[72] Schilmiller A.L., Schauvinhold I., Larson M., Xu R., Charbonneau A.L., Schmidt A., Wilkerson C., Last R.L., Pichersky E. (2009). Monoterpenes in the glandular trichomes of tomato are synthesized from a neryl diphosphate precursor rather than geranyl diphosphate. Proc. Natl. Acad. Sci..

[73] Burke C.C., Wildung M.R., Croteau R. (1999). Geranyl diphosphate synthase: cloning, expression, and characterization of this prenyltransferase as a heterodimer. Proc. Natl. Acad. Sci..

[74] Colby S.M., Alonso W.R., Katahira E.J., Mcgarvey D.J., Croteau R. (1993). 4S-Limonene synthase from the oil glands of spearmint (*Mentha-Spicata*)—cDNA isolation, characterization, and bacterial expression of the catalytically active monoterpene cyclase. J. Biol. Chem..

[75] Lupien S., Karp F., Wildung M., Croteau R. (1999). Regiospecific cytochrome P450 limonene hydroxylases from mint (Mentha) species: cDNA isolation, characterization, and functional expression of (−)-4S-limonene-3-hydroxylase and (−)-4S-limonene-6-hydroxylase. Arch. Biochem. Biophys..

[76] Ringer K.L., Davis E.M., Croteau R. (2005). Monoterpene metabolism. Cloning, expression, and characterization of (-)-isopiperitenol/(-)-carveol dehydrogenase of peppermint and spearmint. Plant Physiol..

[77] Ringer K.L., McConkey M.E., Davis E.M., Rushing G.W., Croteau R. (2003). Monoterpene double-bond reductases of the (-)-menthol biosynthetic pathway: isolation and characterization of cDNAs encoding (-)-isopiperitenone reductase and (+)-pulegone reductase of peppermint. Arch. Biochem. Biophys..

[78] Croteau R., Venkatachalam K.V. (1986). Metabolism of monoterpenes: Demonstration that (+)-cis-isopulegone, not piperitenone, is the key intermediate in the conversion of (−)-isopiperitenone to (+)-pulegone in peppermint (Mentha piperita). Arch. Biochem. Biophys..

[79] Bertea C.M., Schalk M., Karp F., Maffei M., Croteau R. (2001). Demonstration that menthofuran synthase of mint (Mentha) is a cytochrome P450 monooxygenase: Cloning, functional expression, and characterization of the responsible gene. Arch. Biochem. Biophys..

[80] Davis E.M., Ringer K.L., McConkey M.E., Croteau R. (2005). Monoterpene metabolism. Cloning, expression, and characterization of menthone reductases from peppermint. Plant Physiol..

[81] Croteau R.B., Davis E.M., Ringer K.L., Wildung M.R. (2005). (−)-Menthol biosynthesis and molecular genetics. Naturwissenschaften.

[82] McConkey M.E., Gershenzon J., Croteau R.B. (2000). Developmental regulation of monoterpene biosynthesis in the glandular trichomes of peppermint. Plant Physiol..

[83] Rios-Estepa R., Lange B.M. (2007). Experimental and mathematical approaches to modeling plant metabolic networks. Phytochemistry.

[84] Rios-Estepa R., Lange I., Lee J.M., Lange B.M. (2010). Mathematical modeling-guided evaluation of biochemical, developmental, environmental, and genotypic determinants of essential oil composition and yield in peppermint leaves. Plant Physiol..

[85] Rios-Estepa R., Turner G.W., Lee J.M., Croteau R.B., Lange B.M. (2008). A systems biology approach identifies the biochemical mechanisms regulating monoterpenoid essential oil composition in peppermint. Proc. Natl. Acad. Sci..

[86] Khojasteh-Bakht S.C., Chen W., Koenigs L.L., Peter R.M., Nelson S.D. (1999). Metabolism of (*R*)-(+)-pulegone and (*R*)-(+)-menthofuran by human liver cytochrome P-450s: Evidence for formation of a furan epoxide. Drug Metab. Dispos..

[87] Riddle J.M. (1991). Oral contraceptives and early-term abortifacients during classical antiquity and the Middle Ages. Past Present.

[88] Mahmoud S.S., Croteau R.B. (2001). Metabolic engineering of essential oil yield and composition in mint by altering expression of deoxyxylulose phosphate reductoisomerase and menthofuran synthase. Proc. Natl. Acad. Sci. USA.

[89] Devane W., Hanus L., Breuer A., Pertwee R., Stevenson L., Griffin G., Gibson D., Mandelbaum A., Etinger A., Mechoulam R. (1992). Isolation and structure of a brain constituent that binds to the cannabinoid receptor. Science.

[90] Mechoulam R. (1986). The Pharmacohistory of Cannabis Sativa.

[91] Turner J.C., Hemphill J.K., Mahlberg P.G. (1978). Quantitative determination of cannabinoids in individual glandular trichomes of Cannabis sativa L. (Cannabaceae). Am. J. Bot..

[92] Degenhardt F., Stehle F., Kayser O. (2017). The biosynthesis of cannabinoids. Handbook of Cannabis and Related Pathologies.

[93] Fellermeier M., Zenk M.H. (1998). Prenylation of olivetolate by a hemp transferase yields cannabigerolic acid, the precursor of tetrahydrocannabinol. FEBS Lett..

[94] Taura F., Tanaka S., Taguchi C., Fukamizu T., Tanaka H., Shoyama Y., Morimoto S. (2009). Characterization of olivetol synthase, a polyketide synthase putatively involved in cannabinoid biosynthetic pathway. FEBS Lett..

[95] Gagne S.J., Stout J.M., Liu E., Boubakir Z., Clark S.M., Page J.E. (2012). Identification of olivetolic acid cyclase from Cannabis sativa reveals a unique catalytic route to plant polyketides. Proc. Natl. Acad. Sci. USA.

[96] Taura F., Sirikantaramas S., Shoyama Y., Yoshikai K., Shoyama Y., Morimoto S. (2007). Cannabidiolic-acid synthase, the chemotype-determining enzyme in the fiber-type Cannabis sativa. FEBS Lett..

[97] Sirikantaramas S., Morimoto S., Shoyama Y., Ishikawa Y., Wada Y., Shoyama Y., Taura F. (2004). The Gene Controlling Marijuana Psychoactivity: Molecular cloning and heterologous expression of D^1^-tetrahydrocannabinolic acid synthase from Cannabis Sativa L.. J. Biol. Chem..

[98] Taura F., Morimoto S., Shoyama Y., Mechoulam R. (1995). First direct evidence for the mechanism of. DELTA. 1-tetrahydrocannabinolic acid biosynthesis. J. Am. Chem. Soc..

[99] De Meijer E., Hammond K. (2016). The inheritance of chemical phenotype in Cannabis sativa L.(V): Regulation of the propyl-/pentyl cannabinoid ratio, completion of a genetic model. Euphytica.

[100] Flores-Sanchez I.J., Verpoorte R. (2008). Secondary metabolism in cannabis. Phytochem. Rev..

[101] Shoyama Y., Hirano H., Nishioka I. (1984). Biosynthesis of propyl cannabinoid acid and its biosynthetic relationship with pentyl and methyl cannabinoid acids. Phytochemistry.

[102] Matsuda L.A., Lolait S.J., Brownstein M.J., Young A.C., Bonner T.I. (1990). Structure of a cannabinoid receptor and functional expression of the cloned cDNA. Nature.

[103] Herkenham M., Lynn A.B., Little M.D., Johnson M.R., Melvin L.S., de Costa B.R., Rice K.C. (1990). Cannabinoid receptor localization in brain. Proc. Natl. Acad. Sci..

[104] Gutzeit H.O., Ludwig-Müller J. (2014). Plant Natural Products: Synthesis, Biological Functions and Practical Applications.

[105] Pertwee R.G. (2008). The diverse CB1 and CB2 receptor pharmacology of three plant cannabinoids: Δ^9^-tetrahydrocannabinol, cannabidiol and Δ^9^-tetrahydrocannabivarin. Br. J. Pharmacol..

[106] Kaplan B.L., Springs A.E., Kaminski N.E. (2008). The profile of immune modulation by cannabidiol (CBD) involves deregulation of nuclear factor of activated T cells (NFAT). Biochem. Pharmacol..

[107] Friedman D., Devinsky O. (2015). Cannabinoids in the treatment of epilepsy. N. Engl. J. Med..

[108] Barrett M.L., Scutt A.M., Evans F.J. (1986). Cannflavin A and B, prenylated flavones from Cannabis sativa L.. Experientia.

[109] Rea K.A., Casaretto J.A., Al-Abdul-Wahid M.S., Sukumaran A., Geddes-McAlister J., Rothstein S.J., Akhtar T.A. (2019). Biosynthesis of cannflavins A and B from Cannabis sativa L.. Phytochemistry.

[110] Formukong E.A., Evans A.T., Evans F.J. (1988). Analgesic and antiinflammatory activity of constituents of Cannabis sativa L.. Inflammation.

[111] Werz O., Seegers J., Schaible A.M., Weinigel C., Barz D., Koeberle A., Allegrone G., Pollastro F., Zampieri L., Grassi G. (2014). Cannflavins from hemp sprouts, a novel cannabinoid-free hemp food product, target microsomal prostaglandin E2 synthase-1 and 5-lipoxygenase. PharmaNutrition.

[112] Sharifi-Rad J., Sureda A., Tenore G., Daglia M., Sharifi-Rad M., Valussi M., Tundis R., Sharifi-Rad M., Loizzo M., Ademiluyi A. (2017). Biological activities of essential oils: From plant chemoecology to traditional healing systems. Molecules.

[113] Meshnick S.R., Taylor T.E., Kamchonwongpaisan S. (1996). Artemisinin and the antimalarial endoperoxides: From herbal remedy to targeted chemotherapy. Microbiol. Rev..

[114] Nosten F., White N.J. (2007). Artemisinin-Based Combination Treatment of Falciparum Malaria. Am. J. Trop. Med. Hyg..

[115] Tu Y. (2016). Artemisinin—A gift from traditional Chinese medicine to the world (Nobel lecture). Angew. Chem. Int. Edit..

[116] Meshnick S.R. (2002). Artemisinin: Mechanisms of action, resistance and toxicity. Int. J. Parasitol..

[117] O’neill P.M., Barton V.E., Ward S.A. (2010). The molecular mechanism of action of artemisinin—The debate continues. Molecules.

[118] Wu Y. (2002). How might qinghaosu (artemisinin) and related compounds kill the intraerythrocytic malaria parasite? A chemist’s view. Acc. Chem. Res..

[119] Golenser J., Waknine J.H., Krugliak M., Hunt N.H., Grau G.E. (2006). Current perspectives on the mechanism of action of artemisinins. Int. J. Parasitol..

[120] Robert A., Meunier B. (1998). Is alkylation the main mechanism of action of the antimalarial drug artemisinin?. Chem. Soc. Rev..

[121] Asawamabasakda W., Ittarat I., Chang C.-C., McElroy P., Meshnick S.R. (1994). Effects of antimalarials and protease inhibitors on plasmodial hemozoin production. Mol. Biochem. Parasitol..

[122] Haynes R.K., Monti D., Taramelli D., Basilico N., Parapini S., Olliaro P. (2003). Artemisinin Antimalarials Do Not Inhibit Hemozoin Formation. Antimicrob. Agents Chemother..

[123] Cui L., Su X.-Z. (2009). Discovery, mechanisms of action and combination therapy of artemisinin. Expert Rev. Anti-Infect. Ther..

[124] Bouwmeester H.J., Wallaart T.E., Janssen M.H.A., van Loo B., Jansen B.J.M., Posthumus M.A., Schmidt C.O., De Kraker J.-W., König W.A., Franssen M.C.R. (1999). Amorpha-4,11-diene synthase catalyses the first probable step in artemisinin biosynthesis. Phytochemistry.

[125] Mercke P., Bengtsson M., Bouwmeester H.J., Posthumus M.A., Brodelius P.E. (2000). Molecularcloning, expression, and characterization of amorpha-4,11-diene synthase, a key enzyme of artemisinin biosynthesis in Artemisia annua L.. Arch. Biochem. Biophys..

[126] Bertea C., Freije J., Van der Woude H., Verstappen F., Perk L., Marquez V., De Kraker J.-W., Posthumus M., Jansen B., De Groot A. (2005). Identification of intermediates and enzymes involved in the early steps of artemisinin biosynthesis in Artemisia annua. Planta Med..

[127] Teoh K.H., Polichuk D.R., Reed D.W., Nowak G., Covello P.S. (2006). Artemisia annua L.(Asteraceae) trichome-specific cDNAs reveal CYP71AV1, a cytochrome P450 with a key role in the biosynthesis of the antimalarial sesquiterpene lactone artemisinin. FEBS Lett..

[128] Zhang Y., Teoh K.H., Reed D.W., Maes L., Goossens A., Olson D.J., Ross A.R., Covello P.S. (2008). The molecular cloning of artemisinic aldehyde Δ11 (13) reductase and its role in glandular trichome-dependent biosynthesis of artemisinin in Artemisia annua. J. Biol. Chem..

[129] Teoh K.H., Polichuk D.R., Reed D.W., Covello P.S. (2009). Molecular cloning of an aldehyde dehydrogenase implicated in artemisinin biosynthesis in Artemisia annua. Botany.

[130] Le Coz C.J., Ducombs G., Paulsen E., Johansen J.D., Frosch P.J., Lepoittevin J.-P. (2011). Plants and Plant Products. Contact Dermatitis.

[131] Abderrahim O., Martin G.J., Abdelaziz A. (2013). Botanical identification and ethno-medicinal uses of some underground part of medicinal plants collected and traded in Marrakech region. J. Med. Plants Res..

[132] Simmonds P. (1891). The medicinal and other useful plants of Algeria. Am. J. Pharm..

[133] Patkar S.A., Rasmussen U., Diamant B. (1979). On the mechanism of histamine release induced by thapsigargin fromThapsia garganica L.. Agents Actions.

[134] Thastrup O., Cullen P.J., Drøbak B.K., Hanley M.R., Dawson A.P. (1990). Thapsigargin, a tumor promoter, discharges intracellular Ca2+ stores by specific inhibition of the endoplasmic reticulum Ca^2+^-ATPase. Proc. Natl. Acad. Sci. USA.

[135] Sagara Y., Fernandez-Belda F., De Meis L., Inesi G. (1992). Characterization of the inhibition of intracellular Ca2+ transport ATPases by thapsigargin. J. Biol. Chem..

[136] Jiang S., Chow S., Nicotera P., Orrenius S. (1994). Intracellular Ca2+ signals activate apoptosis in thymocytes: Studies using the Ca2+-ATPase inhibitor thapsigargin. Exp. Cell Res..

[137] Ganley I.G., Wong P.-M., Gammoh N., Jiang X. (2011). Distinct autophagosomal-lysosomal fusion mechanism revealed by thapsigargin-induced autophagy arrest. Mol. Cell.

[138] Denmeade S.R., Jakobsen C.M., Janssen S., Khan S.R., Garrett E.S., Lilja H., Christensen S.B., Isaacs J.T. (2003). Prostate-specific antigen-activated thapsigargin prodrug as targeted therapy for prostate cancer. J. Natl. Cancer Inst..

[139] Mahalingam D., Wilding G., Denmeade S., Sarantopoulas J., Cosgrove D., Cetnar J., Azad N., Bruce J., Kurman M., Allgood V. (2016). Mipsagargin, a novel thapsigargin-based PSMA-activated prodrug: Results of a first-in-man phase I clinical trial in patients with refractory, advanced or metastatic solid tumours. Br. J. Cancer.

[140] Mahalingam D., Peguero J., Cen P., Arora S.P., Sarantopoulos J., Rowe J., Allgood V., Tubb B., Campos L. (2019). A Phase II, Multicenter, Single-Arm Study of Mipsagargin (G-202) as a Second-Line Therapy Following Sorafenib for Adult Patients with Progressive Advanced Hepatocellular Carcinoma. Cancers.

[141] Andersen T.B., López C.Q., Manczak T., Martinez K., Simonsen H.T. (2015). Thapsigargin—From Thapsia, L. to Mipsagargin. Molecules.

[142] Pickel B., Drew D.P., Manczak T., Weitzel C., Simonsen H.T., Ro D.-K. (2012). Identification and characterization of a kunzeaol synthase from Thapsia garganica: Implications for the biosynthesis of the pharmaceutical thapsigargin. Biochem. J..

[143] Andersen T.B., Martinez-Swatson K.A., Rasmussen S.A., Boughton B.A., Jørgensen K., Andersen-Ranberg J., Nyberg N., Christensen S.B., Simonsen H.T. (2017). Localization and in-vivo characterization of Thapsia garganica: CYP76AE2 indicates a role in thapsigargin biosynthesis. Plant Physiol..

[144] Nguyen D.T., Göpfert J.C., Ikezawa N., MacNevin G., Kathiresan M., Conrad J., Spring O., Ro D.-K. (2010). Biochemical conservation and evolution of germacrene A oxidase in Asteraceae. J. Biol. Chem..

[145] Møller B.L. (2010). Dynamic metabolons. Science.

[146] Wani M.C., Taylor H.L., Wall M.E., Coggon P., McPhail A.T. (1971). Plant antitumor agents. VI. Isolation and structure of taxol, a novel antileukemic and antitumor agent from Taxus brevifolia. J. Am. Chem. Soc..

[147] Kaspera R., Croteau R. (2006). Cytochrome P450 oxygenases of Taxol biosynthesis. Phytochem. Rev..

[148] Schiff P.B., Fant J., Horwitz S.B. (1979). Promotion of microtubule assembly in vitro by taxol. Nature.

[149] Suffness M., Wall M. (1995). Discovery and Development of Taxol.

[150] McElroy C., Jennewein S. (2018). Taxol^®^ biosynthesis and production: From forests to fermenters. Biotechnology of Natural Products.

[151] Croteau R., Ketchum R.E.B., Long R.M., Kaspera R., Wildung M.R. (2006). Taxol biosynthesis and molecular genetics. Phytochem. Rev..

[152] Guerra-Bubb J., Croteau R., Williams R.M. (2012). The early stages of taxol biosynthesis: An interim report on the synthesis and identification of early pathway metabolites. Nat. Prod. Rep..

[153] Chau M., Croteau R. (2004). Molecular cloning and characterization of a cytochrome P450 taxoid 2α-hydroxylase involved in Taxol biosynthesis. Arch. Biochem. Biophys..

[154] Wildung M.R., Croteau R. (1996). A cDNA clone for taxadiene synthase, the diterpene cyclase that catalyzes the committed step of taxol biosynthesis. J. Biol. Chem..

[155] Jennewein S., Long R.M., Williams R.M., Croteau R. (2004). Cytochrome P450 taxadiene 5α-hydroxylase, a mechanistically unusual monooxygenase catalyzing the first oxygenation step of taxol biosynthesis. Chem. Biol..

[156] Walker K., Schoendorf A., Croteau R. (2000). Molecular cloning of a taxa-4 (20), 11 (12)-dien-5α-ol-O-acetyl transferase cDNA from Taxus and functional expression in Escherichia coli. Arch. Biochem. Biophys..

[157] Ondari M.E., Walker K.D. (2008). The taxol pathway 10-O-acetyltransferase shows regioselective promiscuity with the oxetane hydroxyl of 4-deacetyltaxanes. J. Am. Chem. Soc..

[158] Walker K., Croteau R. (2000). Molecular cloning of a 10-deacetylbaccatin III-10-O-acetyl transferase cDNA from Taxus and functional expression in Escherichia coli. Proc. Natl. Acad. Sci. USA.

[159] Walker K.D., Klettke K., Akiyama T., Croteau R. (2004). Cloning, heterologous expression, and characterization of a phenylalanine aminomutase involved in Taxol biosynthesis. J. Biol. Chem..

[160] Ramírez-Estrada K., Altabella T., Onrubia M., Moyano E., Notredame C., Osuna L., Vanden Bossche R., Goossens A., Cusido R.M., Palazon J. (2016). Transcript profiling of jasmonate-elicited Taxus cells reveals a β-phenylalanine-CoA ligase. Plant Biotechnol. J..

[161] Walker K., Fujisaki S., Long R., Croteau R. (2002). Molecular cloning and heterologous expression of the C-13 phenylpropanoid side chain-CoA acyltransferase that functions in Taxol biosynthesis. Proc. Natl. Acad. Sci..

[162] Walker K., Long R., Croteau R. (2002). The final acylation step in taxol biosynthesis: Cloning of the taxoid C13-side-chain N-benzoyltransferase from Taxus. Proc. Natl. Acad. Sci. USA.

[163] Dueber M.T., Adolf W., West C.A. (1978). Biosynthesis of the Diterpene Phytoalexin Casbene. Plant Physiol..

[164] Mau C., West C.A. (1994). Cloning of casbene synthase cDNA: Evidence for conserved structural features among terpenoid cyclases in plants. Proc. Natl. Acad. Sci..

[165] Kirby J., Nishimoto M., Park J.G., Withers S.T., Nowroozi F., Behrendt D., Rutledge E.J.G., Fortman J.L., Johnson H.E., Anderson J.V. (2010). Cloning of casbene and neocembrene synthases from Euphorbiaceae plants and expression in Saccharomyces cerevisiae. Phytochemistry.

[166] Kulkosky J., Culnan D.M., Roman J., Dornadula G., Schnell M., Boyd M.R., Pomerantz R.J. (2001). Prostratin: Activation of latent HIV-1 expression suggests a potential inductive adjuvant therapy for HAART. Blood.

[167] Szallasi A., Blumberg P. (1989). Resiniferatoxin, a phorbol-related diterpene, acts as an ultrapotent analog of capsaicin, the irritant constituent in red pepper. Neuroscience.

[168] Luo D., Callari R., Hamberger B., Wubshet S.G., Nielsen M.T., Andersen-Ranberg J., Hallström B.M., Cozzi F., Heider H., Møller B.L. (2016). Oxidation and cyclization of casbene in the biosynthesis of Euphorbia factors from mature seeds of Euphorbia lathyris L.. Proc. Natl. Acad. Sci. USA.

[169] Siller G., Gebauer K., Welburn P., Katsamas J., Ogbourne S.M. (2009). PEP005 (ingenol mebutate) gel, a novel agent for the treatment of actinic keratosis: Results of a randomized, double-blind, vehicle-controlled, multicentre, phase IIa study. Australas. J. Dermatol..

[170] Kedei N., Lundberg D.J., Toth A., Welburn P., Garfield S.H., Blumberg P.M. (2004). Characterization of the interaction of ingenol 3-angelate with protein kinase C. Cancer Res..

[171] Niedel J.E., Kuhn L.J., Vandenbark G. (1983). Phorbol diester receptor copurifies with protein kinase C. Proc. Natl. Acad. Sci. USA.

[172] Emerit I., Cerutti P.A. (1981). Tumour promoter phorbol-12-myristate-13-acetate induces chromosomal damage via indirect action. Nature.

[173] Anderson L., Schmieder G.J., Werschler W.P., Tschen E.H., Ling M.R., Stough D.B., Katsamas J. (2009). Randomized, double-blind, double-dummy, vehicle-controlled study of ingenol mebutate gel 0.025% and 0.05% for actinic keratosis. J. Am. Acad. Dermatol..

[174] Hampson P., Chahal H., Khanim F., Hayden R., Mulder A., Assi L.K., Bunce C.M., Lord J.M. (2005). PEP005, a selective small-molecule activator of protein kinase C, has potent antileukemic activity mediated via the delta isoform of PKC. Blood.

[175] Shi Q.-W., Su X.-H., Kiyota H. (2008). Chemical and pharmacological research of the plants in genus Euphorbia. Chem. Rev..

[176] Uemura D., Nobuhara K., Nakayama Y., Shizuri Y., Hirata Y. (1976). The structure of new lathyrane diterpenes, jolkinols a, b, c, and d, from Euphorbia jolkini Boiss. Tetrahedron Lett..

[177] Mithöfer A., Boland W. (2012). Plant defense against herbivores: Chemical aspects. Annu. Rev. Plant Biol..

[178] Gershenzon J. (1994). Metabolic costs of terpenoid accumulation in higher plants. J. Chem. Ecol..

